# The correlation between changes in gray matter microstructure and cerebral blood flow in Alzheimer’s disease

**DOI:** 10.3389/fnagi.2023.1205838

**Published:** 2023-06-02

**Authors:** Xiaoxi Niu, Ying Guo, Zhongyu Chang, Tongtong Li, Yuanyuan Chen, Xianchang Zhang, Hongyan Ni

**Affiliations:** ^1^First Central Clinical College, Tianjin Medical University, Tianjin, China; ^2^Department of Radiology and Tianjin Key Laboratory of Functional Imaging, Tianjin Medical University General Hospital, Tianjin, China; ^3^Department of Radiology, Affiliated Hospital of Hebei University, Baoding, China; ^4^Tianjin International Joint Research Center for Neural Engineering, Academy of Medical Engineering and Translational Medicine, Tianjin University, Tianjin, China; ^5^MR Collaboration, Siemens Healthcare Ltd., Beijing, China; ^6^Department of Radiology, Tianjin First Central Hospital, Tianjin, China

**Keywords:** microstructure, cerebral blood flow (CBF), diffusion kurtosis imaging (DKI), arterial spin labeling (ASL), Alzheimer’s disease, mild cognitive impairment (MCI), gray matter

## Abstract

**Objective:**

To investigate the relationship between changes in cerebral blood flow (CBF) and gray matter (GM) microstructure in Alzheimer’s disease (AD) and mild cognitive impairment (MCI).

**Methods:**

A recruited cohort of 23 AD patients, 40 MCI patients, and 37 normal controls (NCs) underwent diffusional kurtosis imaging (DKI) for microstructure evaluation and pseudo-continuous arterial spin labeling (pCASL) for CBF assessment. We investigated the differences in diffusion- and perfusion-related parameters across the three groups, including CBF, mean diffusivity (MD), mean kurtosis (MK), and fractional anisotropy (FA). These quantitative parameters were compared using volume-based analyses for the deep GM and surface-based analyses for the cortical GM. The correlation between CBF, diffusion parameters, and cognitive scores was assessed using Spearman coefficients, respectively. The diagnostic performance of different parameters was investigated with k-nearest neighbor (KNN) analysis, using fivefold cross-validation to generate the mean accuracy (mAcc), mean precision (mPre), and mean area under the curve (mAuc).

**Results:**

In the cortical GM, CBF reduction primarily occurred in the parietal and temporal lobes. Microstructural abnormalities were predominantly noted in the parietal, temporal, and frontal lobes. In the deep GM, more regions showed DKI and CBF parametric changes at the MCI stage. MD showed most of the significant abnormalities among all the DKI metrics. The MD, FA, MK, and CBF values of many GM regions were significantly correlated with cognitive scores. In the whole sample, the MD, FA, and MK were associated with CBF in most evaluated regions, with lower CBF values associated with higher MD, lower FA, or lower MK values in the left occipital lobe, left frontal lobe, and right parietal lobe. CBF values performed best (mAuc = 0.876) for distinguishing the MCI from the NC group. Last, MD values performed best (mAuc = 0.939) for distinguishing the AD from the NC group.

**Conclusion:**

Gray matter microstructure and CBF are closely related in AD. Increased MD, decreased FA, and MK are accompanied by decreased blood perfusion throughout the AD course. Furthermore, CBF values are valuable for the predictive diagnosis of MCI and AD. GM microstructural changes are promising as novel neuroimaging biomarkers of AD.

## Introduction

Alzheimer’s disease (AD) is a neurodegenerative disorder characterized by extracellular accumulation of amyloid β protein (Aβ) plaques and intracellular neurofibrillary tau tangles (Guo et al., 2020). Studies within the last few decades provide growing evidence that cerebral vascular dysfunction in various molecular pathways plays a central role in AD pathogenesis. Due to blood–brain barrier (BBB) breakdown, the accumulation of excess neurotoxic Aβ occurs, resulting in impaired BBB function and cerebral hypoperfusion (Chakraborty et al., 2017; Yamazaki and Kanekiyo, 2017). In addition, restricted Aβ clearance further damages surrounding neurons and neurites, leading to microstructural gray matter (GM) loss, as in the hippocampus with associated cognitive changes (Wang et al., 2018). Given that brain microstructural and cerebral blood flow (CBF) changes occur years before the onset of clinical symptoms, efforts to detect these alterations early could significantly enhance our ability to diagnose AD sooner.

Diffusion kurtosis imaging (DKI) is an MRI technique that extends conventional diffusion tensor imaging (DTI) by estimating the kurtosis of the water diffusion probability distribution function, allowing for subtle pathology quantification in gray and white matter architecture (Jensen et al., 2005). DKI provides kurtosis metrics such as mean kurtosis (MK) related to the non-Gaussianity of water diffusion for reflecting microstructure complexity. In addition, diffusion parameters such as fractional anisotropy (FA) and mean diffusivity (MD) are also acquired from DKI, symbols of neuronal status, including axonal, dendritic, and synaptic integrity. Although DKI applications in AD and mild cognitive impairment (MCI) have been largely performed to probe alterations in white matter, they have great potential for detecting GM microstructural changes.

One study showed that microstructural abnormalities in cortical GM occurred primarily in the parietal and frontal lobes in AD (Gong et al., 2017). Moreover, some research suggested that cortical MD was sensitive enough to predict progression to MCI (Wang et al., 2018; Rodriguez-Vieitez et al., 2021). Arterial spin labeling (ASL) is a non-invasive technique that labels arterial blood water as an endogenous contrast tracer to measure CBF at the tissue level (Hernandez-Garcia et al., 2019). The diagnostic performance detected by ASL was similar to that identified by single photon emission computed tomography (SPECT) (Johnson et al., 2005). Hence, ASL has become a fundamental CBF imaging technique in brain research. In addition, some studies suggested that CBF can serve as a reliable hallmark in the early diagnosis of AD and MCI (Hays et al., 2016; Korte et al., 2020; Camargo and Wang, 2021).

With combined cerebral microstructure and CBF, Alisch et al. (2021) found that lower microstructural integrity was associated with lower CBF in the choroid plexus. This founding suggested that adequate CBF is paramount for nutrient and oxygen delivery and the maintenance of tissue integrity. Moreover, Bouhrara et al. (2022) found that FA and MD were associated with CBF in most regions evaluated, including the frontal and parietal white matter lobes in healthy adults. All these findings indicated that the CBF changes might affect cerebral tissue health. However, Bouhrara’s study only focused on cohorts of cognitively unimpaired subjects, and their results about association between CBF and GM microstructure were mostly not significant in most regions, likely due to the limitation of DTI to capture changes in GM as compared to DKI technique. Furthermore, the association between GM microstructural integrity and perfusion in individuals with cognitive impairment has currently received little consideration. So it is necessary that we combined DKI and pseudo-continuous arterial spin labeling (pCASL) to investigate further associations established in cognitive impairments.

Given the impairment of neurovascular coupling in AD pathogenesis, there may have a potential link between them. Therefore, the specific aims of this study were to analyze abnormalities of the GM microstructure and CBF and determine the association of these changes with cognitive function, to investigate the relationship between microstructural and perfusion parameters in combined DKI and ASL assessment in early AD patients, and to explore the diagnostic performance of MD, FA, MK, and CBF values in AD and MCI patients.

## Materials and methods

### Subjects

The study was approved by the hospital ethics committee, and all subjects signed informed consent. Twenty-three AD patients, 40 MCI patients, and 37 healthy age- and sex-matched controls were recruited in our hospital from September 2021 to December 2022. All subjects underwent MRI examinations and cognitive evaluations with psychological tests such as the mini-mental state examination (MMSE), Montreal cognitive assessment (MoCA), Clinical Dementia Rating (CDR) Scale, activities of daily living (ADL) assessment, Hachinski Ischemic Scale (HIS), and the Hamilton Depression Rating Scale (HAMD).

Patients met the criteria for AD based on the National Institute on Aging and Alzheimer’s Association (NIA-AA) (McKhann et al., 2011) and for MCI using Petersen’s criteria (Petersen et al., 2018). The exclusion criteria were as follows: (1) patients with brain diseases other than AD or MCI; (2) severe cerebrovascular diseases and HIS ≥4; (3) patients with a psychiatric disorder or severe head trauma history; (4) poor image quality in post-processing; or (5) left-handedness.

### MRI acquisition

All the participants underwent MR scans on a 3T MAGNETOM Prisma MR scanner (Siemens Healthcare, Erlangen, Germany) equipped with a 64-channel head-neck coil. In addition, 3D-pCASL images were acquired using the following parameters: TR/TE = 4,100/36.68 ms, FOV = 240 mm × 240 mm, matrix = 96 × 96, slice thickness = 3 mm, multiple post-labeling delay time = 500/1,000/1,500/2,000/2,500 ms, flip angle = 120°, and 12 pairs of label and control images with a scan time of 7 min and 11 s. Furthermore, diffusion-weighted images were obtained with a spin-echo echo-planar imaging (SE-EPI) sequence. The DKI parameters were TR/TE = 3,800/72 ms, FOV = 220 mm × 220 mm, voxel = 2.0 mm × 2.0 mm × 2.2 mm, matrix = 110 × 110, slices = 60, simultaneous multi-slice acquisition (SMS) acceleration factor = 2, *b* = 0/2,000/,3000 s/mm^2^, and directions = 64. The parameters for 3D T1-weighted imaging acquisition were TR/TE = 1,550/2.98 ms, flip angle = 9°, FOV = 256 mm × 256 mm, voxel = 1.0 mm × 1.0 mm × 1.0 mm, and slices = 176.

### Image analysis

#### ASL image processing

Raw ASL data were analyzed with CereFlow software programs.^1^ Motion correction was performed on all ASL images. Pairwise subtraction was used between the label and control images, followed by averaging to generate the mean image. Quantitative CBF maps were calculated using a previously reported model (Hernandez-Garcia et al., 2019). Then, registration to the AAL atlas was performed on CBF maps to calculate the CBF values of every ROI.

#### DKI image processing

First, all raw diffusion images were converted to the Neuroimaging Informatics Technology Initiative (NIFTI) format. Then, a rigid body transformation between the *b* = 0 image and all the diffusion-weighted acquisition was applied to mitigate motion effects. After correcting for movement, non-brain tissue was removed using the Brain Extraction Tool with the FSL toolbox (Smith et al., 2004) (Oxford Centre for Functional MRI of the brain).^2^ Finally, Diffusional Kurtosis Estimator (DKE) software (Tabesh et al., 2011)^3^ was used to calculate the MK, FA, and MD maps by loading 4D NIFTI diffusion images.

#### FreeSurfer processing

The segmentation of T1-weighted images was performed with the FreeSurfer package^4^ using the recon-all pipeline that has been described in a previous publication (Montal et al., 2018). This processing comprises motion correction and a skull strip, talairach transform computation, automated segmentation of subcortical white matter, and deep GM structures.

We used a surface-based approach to process cortical diffusion and perfusion images because the commonly used voxel-based morphometry approach has defects in GM analyses, where partial volume effects may make cortical parameter measurements that are biased due to the contribution from the CSF and white matter signal on the GM voxels. The detailed surface-based DKI steps were as follows: (1) the skull-stripped b0 diffusion images were co-registered to the segmented structural T1-weighted volume using the *bbregister* command, a boundary-based registration algorithm in FreeSurfer (Greve and Fischl, 2009). All registrations were visually inspected to detect and correct processing errors in the registration; (2) DKI volumes, including MD, FA, and MK, were projected to each individual’s surface space generated during the cortical segmentation. At each vertex, cortical DKI maps were sampled at the midpoint along the normal vector between white and pial surfaces using the *mri_vol2surf* command (Greve et al., 2014); and (3) All maps were normalized to a standard surface template (fsaverage) and smoothed with a 15-mm full-width half-maximum (FWHM) Gaussian kernel.

The process of surface-based perfusion images was similar to the described approach. First, a boundary-based registration of the CBF and M0 volumes to the T1-weighted volume was performed, followed by projection on an averaged surface. Then, all maps were smoothed with a 15-mm FWHM Gaussian kernel. In addition, cortical Desikan-Killiany atlases were used to extract DKI and CBF GM parameters.

All the maps analysis were applied a partial volume effect correction in order to improve the accuracy of measurements. After motion correction, maps estimation and registration, the contribution of the CSF in each voxel, as segmented by FreeSurfer’s *gtmseg*, was computed to correct the diffusion – and perfusion – maps in the cortical GM (Greve et al., 2016). Then, *mri_gtmpvc* command was used to carry out partial volume correction. The maps without contamination of CSF were performed further process.

### Statistical analyses

The demographic data and cognitive scores of all subjects were analyzed with SPSS 26.0 statistical software (IBM Corp., Armonk, NY, USA). Normal distribution and homogeneity of variance were applied to all quantitative data. The subjects’ age, education, MMSE, and MoCA differences were tested with Kruskal–Wallis, and sex differences were tested with the chi-squared test in the AD, MCI, and normal control (NC) groups.

We first performed group analyses for MD, FA, MK, and CBF of the cortical GM with general linear models, as implemented in FreeSurfer [family-wise error (FWE), *p* < 0.05] between the NC, MCI, and AD stages. In this work, we used the Monte Carlo simulation with 5,000 repeats to generate a distribution of maximum cluster size. Then, ROI parameters in the deep GM were compared using ANOVA, and the Bonferroni correction was performed in *post hoc* pairwise comparisons between the sub-groups. The correlation between DKI parameters, CBF, and cognitive scores was analyzed with Spearman’s correlation. Simple linear regression adjusted age and sex was applied to analyze the association between GM CBF and diffusion indices calculated in eight defined GM ROIs.

Finally, we used k-nearest neighbor (KNN) with the Sklearn library in Python to establish classification model and investigate the diagnostic power of DKI and CBF parameters for MCI and AD dementia. For this analysis, we first selected ROIs with f_classif method as predicators for the parameters of each metric, *f*-value was higher, the diagnostic value was higher. For the classification of AD and NC, the most important ROIs were the left inferior temporal, the left isthmus cingulate and the left lateral occipital for CBF; the most important ROIs were bilateral hippocampus and the right inferior temporal for MD; the most important ROIs were the left entorhinal, the right precentral and the right rostral middle frontal for FA; the most important ROIs were the left caudal anterior cingulate, the left posterior cingulate and the right caudal middle frontal for MK. For the classification of MCI and NC, the most important ROIs were the left entorhinal, the left inferior parietal and the left inferior temporal for CBF; the most important ROIs were the left entorhinal, the right middle temporal and the right superior parietal for MD; the most important ROIs were the left lateral orbital frontal, the right precentral and the left putamen for FA; the most important ROIs were the right fusiform, the right lateral occipital and the left putamen for MK. We then generated mean area under the curve (mAuc), mean accuracy (mAcc), and mean precision (mPre) by running *cross_val_score* command (*cv* = 5) for each diagnostic group.

## Results

### Demographics

Demographic data and neuropsychological score results of the three groups are shown in Table 1. There were no significant differences in sex (*p* = 0.304), age (*p* = 0.754), or education duration (*p* = 0.135) in the AD, MCI, or NC groups. However, there were significant differences in the MMSE (*p* < 0.001) and MoCA scores (*p* < 0.001) in the three groups.

**TABLE 1:** Demographic data and neuropsychological scores analyses.

Characteristic	AD (*n* = 23)	MCI (*n* = 40)	NC (*n* = 37)	*p*-value
Males, *n* (%)	6 (26.09)	15 (37.5)	17 (45.95)	0.304
Age, median (IQR)	72 (63, 81)	69 (67, 75.8)	69 (66.5, 74)	0.754
Education, median (IQR)	11 (9,12)	9 (8, 12)	11 (9, 15.5)	0.135
MMSE, median (IQR)	12 (9, 19)^ab^	24 (22.3, 27)^a^	29 (28, 30)	<0.001
MoCA, median (IQR)	8 (7, 15)^ab^	19 (17.3, 21.8)^a^	27 (26, 28.5)	<0.001

A Chi-squared test was performed for sex comparisons; the Kruskal–Wallis test was used to compare age, education, MMSE, and MoCA scores, and the results are presented as medians (lower quartile ∼ upper quartile). MMSE, mini-mental state examination; MoCA, Montreal cognitive assessment; AD, Alzheimer’s disease; MCI, mild cognitive impairment; NC, normal control.

^a^Significantly different compared with the NC group.

^b^Significantly different compared with the MCI group.

### Abnormalities in DKI metrics of the cortical and deep gray matter

**FIGURE 1:**
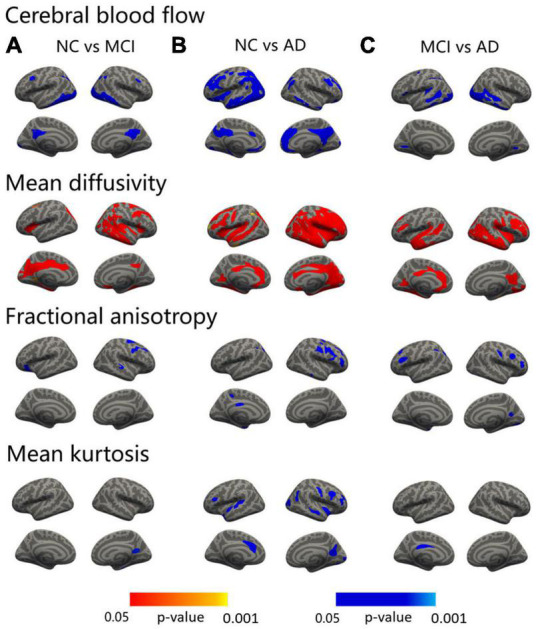
Surface-based statistical map showing the clusters with significant abnormalities in CBF and DKI metrics on the AD continuum. The differences in the CBF and DKI metrics in the NC vs. MCI **(A)**, NC vs. AD **(B)**, and MCI vs. AD **(C)**. Clusters survived correction for multiple comparisons implemented in FreeSurfer by Monte Carlo simulation with 5,000 repeats, with the family-wise error correction settled at *p* < 0.05. The blue color represents decreased values, and the red color represents increased values.

Compared with the NC, the MCI patients showed areas of increased MD in the precuneus, entorhinal, superior parietal, isthmus cingulate, pars opercularis, and caudal middle frontal lobe in the left hemisphere, and middle temporal, fusiform, precuneus, postcentral, precentral, rostral middle frontal, superior frontal, medial orbitofrontal, lateral orbitofrontal, superior parietal, inferior parietal, and pars opercularis in the right hemisphere (Figure 1A, second row and Supplementary Table 1). The AD patients revealed a widespread increased pattern that included the precuneus, insula, precentral, caudal middle frontal, rostral middle frontal, superior parietal, inferior parietal, middle temporal, and supramarginal regions in the left hemisphere, and medial orbitofrontal, inferior temporal, superior parietal, and lateral occipital regions in the right hemisphere (Figure 1B, second row and Supplementary Table 2). Compared with the MCI, the MD values of the bilateral fusiform, bilateral rostral middle frontal, left posterior cingulate, left lingual, left superior frontal, right insula, right precuneus, right inferior temporal, right caudal middle frontal, and right inferior parietal regions were significantly higher in the AD group (Figure 1C, second row and Supplementary Table 3).

The MCI patients displayed regions of decreased FA compared to the NC in the left lateral orbitofrontal, right precentral, and right superior temporal areas (Figure 1A, third row and Supplementary Table 1). The AD patients showed a decreased FA in the superior parietal, inferior parietal, precuneus, entorhinal, and posterior cingulate regions in the left hemisphere and in the pars triangularis, precentral, middle temporal, superior frontal, and rostral middle frontal areas in the right hemisphere (Figure 1B, third row and Supplementary Table 2). Compared with the MCI patients, the FA values of the bilateral rostral middle frontal, left temporal pole, left supramarginal, left superior parietal, right precuneus, right postcentral, right caudal middle frontal, and right lateral occipital regions were significantly lower in the AD group (Figure 1C, third row and Supplementary Table 3).

Significant decreases in MK were shown between the MCI and NC groups in the right precuneus and fusiform regions (Figure 1A, fourth row and Supplementary Table 1). As in the AD versus NC comparisons, decreases in MK were noted in the temporal pole, superior temporal, pars opercularis, and caudal anterior cingulate regions in the left hemisphere and in the precuneus, precentral, insula, caudal middle frontal, rostral middle frontal, superior temporal, lateral occipital, inferior parietal, and pericalcarine areas in the right hemisphere (Figure 1B, fourth row and Supplementary Table 2). In addition, compared with the MCI group, the MK value of the left posterior cingulate was significantly lower in the AD group (Figure 1C, fourth row and Supplementary Table 3).

**FIGURE 2:**
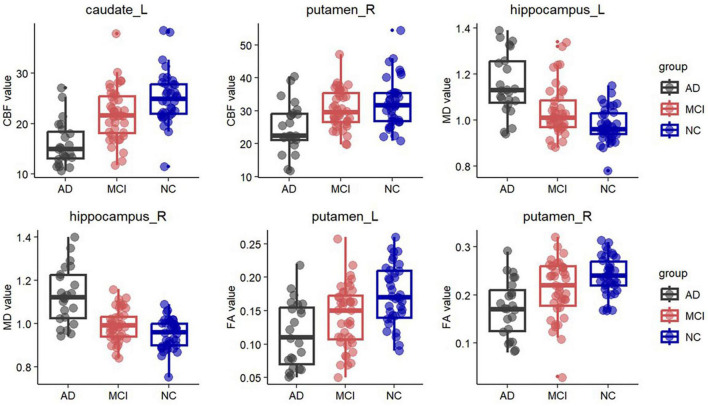
Box plots of fractional anisotropy (FA) in the bilateral putamen, cerebral blood flow (CBF) in the left caudate and right putamen, and mean diffusivity (MD) in the bilateral hippocampus. Significantly lower FA was observed in the bilateral putamen in the AD group and in the left putamen in the MCI group. In the left caudate and right putamen, significantly lower CBF was observed in the AD group. Significantly higher MD was observed in the bilateral hippocampus in the AD group and in the left hippocampus in the MCI group.

In the deep GM, MD showed the most regions with significant abnormalities in the bilateral amygdala, putamen, and left hippocampus at the MCI stage compared to the NC. FA also revealed changes in the left putamen. In addition to the bilateral amygdala, putamen, and hippocampus, significant differences in MD were shown between the AD and NC groups in the bilateral globus pallidus and left thalamus. Abnormalities of FA occurred in the bilateral putamen, right amygdala, and right thalamus at the AD stage. Similarly, compared with the MCI group, the MD value was significantly increased in AD patients, including the bilateral hippocampus, amygdala, putamen, globus pallidus, and the left thalamus. The FA value was significantly decreased in AD, including in the right putamen. However, no significant changes were found in MK (Table 2 and Figure 2).

**TABLE 2:** Group differences of regional CBF and DKI metrics in deep gray matter between NC and MCI/AD.

		NC	MCI	AD	F	*p*-value
**CBF**						
Hippocampus	Left	35.953 (33.426, 38.479)	34.580 (32.370, 36.790)	32.220 (28.667, 35.773)	1.773	0.175
	Right	34.550 (32.096, 37.005)	32.921 (30.800, 35.043)	29.752 (26.535, 32.968)^a^	3.262	0.043
Amygdala	Left	34.806 (32.467, 37.146)	35.849 (33.223, 38.476)	36.893 (32.818, 40.968)	0.478	0.621
	Right	34.623 (32.221, 37.026)	34.331 (32.129, 36.533)	34.660 (29.571, 39.749)	0.016	0.984
Caudate	Left	24.445 (23.113, 25.778)	22.591 (20.555, 24.628)	15.958 (14.049, 17.867)^ab^	19.957	**<0.001**
	Right	24.840 (23.322, 26.358)	22.845 (20.911, 24.779)	19.919 (16.846, 22.992)^a^	5.034	0.008
Putamen	Left	31.360 (29.555, 33.165)	30.612 (28.492, 32.732)	26.069 (21.705, 30.433)^a^	4.209	0.018
	Right	31.013 (29.301, 32.725)	30.818 (28.635, 33.002)	25.013 (21.738, 28.288)^ab^	7.432	0.001
Globus pallidus	Left	33.555 (30.746, 36.364)	33.953 (31.594, 36.312)	31.211 (28.510, 33.912)	1.052	0.353
	Right	34.383 (31.388, 37.377)	34.007 (31.821, 36.193)	29.830 (27.433, 32.228)	3.093	0.050
Thalamus	Left	40.413 (37.765, 43.061)	37.477 (34.940, 40.015)	37.100 (33.744, 40.456)	1.770	0.176
	Right	41.420 (38.350, 44.490)	38.462 (35.606, 41.317)	39.175 (34.362, 43.988)	0.959	0.387
**MD**						
Hippocampus	Left	0.976 (0.952, 1.000)	1.042 (1.006, 1.077)^a^	1.153 (1.096, 1.210)^ab^	20.338	**<0.001**
	Right	0.950 (0.927, 0.973)	0.991 (0.967, 1.015)	1.129 (1.073, 1.185)^ab^	30.353	**<0.001**
Amygdala	Left	1.211 (1.183, 1.239)	1.289 (1.245, 1.333)^a^	1.380 (1.322, 1.439)^ab^	14.272	**<0.001**
	Right	1.042 (1.023, 1.061)	1.121 (1.088, 1.155)^a^	1.249 (1.182, 1.316)^ab^	27.380	**<0.001**
Caudate	Left	0.929 (0.872, 0.985)	1.043 (0.962, 1.124)	0.906 (0.788, 1.023)	3.494	0.034
	Right	0.937 (0.883, 0.990)	1.006 (0.930, 1.081)	0.910 (0.812, 1.008)	1.851	0.163
Putamen	Left	1.502 (1.410, 1.594)	1.751 (1.643, 1.859)^a^	2.029 (1.884, 2.174)^ab^	19.948	**<0.001**
	Right	1.184 (1.113, 1.254)	1.381 (1.264, 1.499)^a^	1.676 (1.514, 1.837)^ab^	16.767	**<0.001**
Globus pallidus	Left	0.796 (0.773, 0.820)	0.815 (0.801, 0.830)	0.876 (0.820, 0.932)^ab^	7.235	0.001
	Right	0.836 (0.812, 0.859)	0.875 (0.843, 0.908)	0.906 (0.884, 0.929)^ab^	5.554	0.005
Thalamus	Left	0.741 (0.723, 0.759)	0.761 (0.746, 0.775)	0.806 (0.765, 0.847)^ab^	7.666	0.001
	Right	0.773 (0.749, 0.796)	0.800 (0.770, 0.830)	0.808 (0.778, 0.838)	1.698	0.188
**MK**						
Hippocampus	Left	0.713 (0.687, 0.739)	0.725 (0.705, 0.745)	0.710 (0.665, 0.754)	0.365	0.695
	Right	0.790 (0.761, 0.819)	0.799 (0.777, 0.820)	0.760 (0.716, 0.805)	1.550	0.218
Amygdala	Left	0.609 (0.599, 0.619)	0.609 (0.600, 0.617)	0.631 (0.562, 0.700)	0.660	0.519
	Right	0.616 (0.603, 0.629)	0.613 (0.606, 0.621)	0.632 (0.569, 0.695)	0.507	0.604
Caudate	Left	0.478 (0.456, 0.500)	0.493 (0.475, 0.512)	0.477 (0.442, 0.512)	0.663	0.517
	Right	0.515 (0.491, 0.540)	0.538 (0.518, 0.557)	0.520 (0.485, 0.554)	1.068	0.348
Putamen	Left	0.590 (0.565, 0.616)	0.545 (0.522, 0.567)	0.544 (0.423, 0.665)	1.102	0.336
	Right	0.673 (0.641, 0.705)	0.639 (0.610, 0.668)	0.622 (0.499, 0.744)	0.853	0.429
Globus pallidus	Left	0.712 (0.676, 0.748)	0.737 (0.727, 0.747)	0.747 (0.727, 0.768)	2.061	0.133
	Right	0.695 (0.660, 0.730)	0.704 (0.695, 0.713)	0.725 (0.697, 0.753)	1.231	0.297
Thalamus	Left	0.872 (0.831, 0.912)	0.894 (0.869, 0.919)	0.887 (0.858, 0.915)	0.554	0.577
	Right	0.804 (0.761, 0.848)	0.823 (0.801, 0.844)	0.858 (0.830, 0.887)	2.265	0.109
**FA**						
Hippocampus	Left	0.256 (0.236, 0.277)	0.264 (0.243, 0.284)	0.257 (0.235, 0.280)	0.165	0.848
	Right	0.350 (0.324, 0.376)	0.352 (0.328, 0.376)	0.332 (0.310, 0.355)	0.606	0.547
Amygdala	Left	0.144 (0.139, 0.149)	0.136 (0.130, 0.142)	0.134 (0.127, 0.141)	3.235	0.044
	Right	0.164 (0.159, 0.169)	0.160 (0.154, 0.165)	0.151 (0.144, 0.158)^a^	5.190	0.007
Caudate	Left	0.117 (0.111, 0.123)	0.116 (0.110, 0.121)	0.113 (0.105, 0.122)	0.341	0.712
	Right	0.141 (0.134, 0.148)	0.137 (0.130, 0.145)	0.131 (0.123, 0.139)	1.497	0.229
Putamen	Left	0.175 (0.161, 0.190)	0.141 (0.126, 0.157)^a^	0.114 (0.093, 0.136)^a^	12.689	**<0.001**
	Right	0.240 (0.227, 0.253)	0.212 (0.193, 0.232)	0.173 (0.147, 0.199)^ab^	11.302	**<0.001**
Globus pallidus	Left	0.307 (0.299, 0.315)	0.320 (0.311, 0.329)	0.323 (0.311, 0.334)	3.407	0.037
	Right	0.293 (0.286, 0.300)	0.293 (0.286, 0.301)	0.300 (0.292, 0.309)	0.986	0.377
Thalamus	Left	0.329 (0.315, 0.344)	0.347 (0.333, 0.361)	0.351 (0.334, 0.369)	2.485	0.089
	Right	0.262 (0.251, 0.274)	0.276 (0.263, 0.290)	0.300 (0.282, 0.318)^a^	6.502	0.002

Numbers are shown in adjusted means with 95% confidence intervals. AD, Alzheimer’s disease; MCI, mild cognitive impairment; NC, normal control; CBF, cerebral blood flow; MD, mean diffusivity; MK, mean kurtosis; FA, fractional anisotropy.

^a^Significantly different compared with the NC group.

^b^Significantly different compared with the MCI group.

The bold values mean *p* < 0.001 and significant difference in groups.

### Abnormalities of CBF in the cortical and deep gray matter

Compared with the NC, the MCI patients showed areas of decreased CBF in the bilateral inferior temporal, isthmus cingulate, caudal middle frontal, left lateral occipital, superior parietal, and the right inferior parietal regions (Figure 1A, first row and Supplementary Table 1). As in the AD vs. NC comparisons, decreases in CBF were noted in the bilateral inferior temporal, lateral orbital frontal, medial orbitofrontal, parahippocampus, the left caudal middle frontal, isthmus cingulate, precentral, caudal anterior cingulate, entorhinal, superior temporal, lateral occipital, right precuneus, rostral middle frontal, inferior parietal, temporal pole, inferior parietal, and pericalcarine regions (Figure 1B, first row and Supplementary Table 2). Compared with the MCI, the CBF values of the left lateral occipital, superior temporal, inferior temporal, lingual, supramarginal, inferior parietal, precentral, caudal middle frontal, right fusiform, middle temporal, and lingual regions were significantly lower in the AD group (Figure 1C, first row and Supplementary Table 3).

In the deep GM, CBF showed significant decreases in the bilateral caudate, putamen, and right hippocampus at the AD stage compared to the NC. Compared with the MCI, the CBF values of the left caudate and right putamen were significantly lower in the AD group. There is no significant difference between the MCI and NC groups (Table 2 and Figure 2).

### Correlations between imaging parameters and neuropsychological scores

**FIGURE 3:**
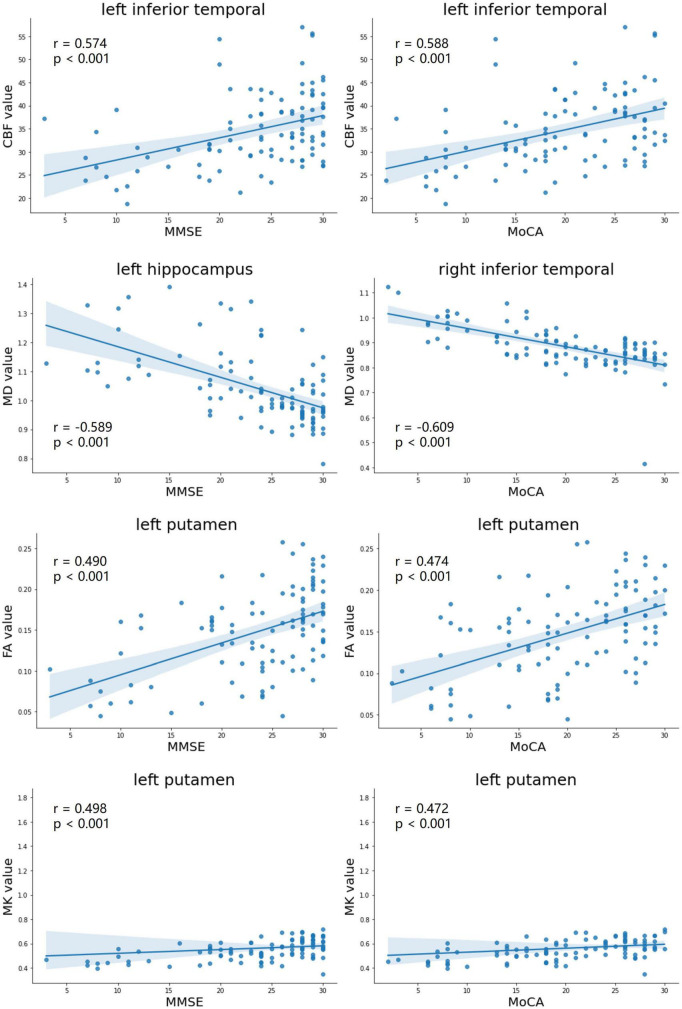
Correlation analyses with maximal correlation coefficients between DKI-derived parameters (MD, FA, and MK), CBF, and neuropsychological testing scores (MMSE scores, MoCA scores). MD, mean diffusivity; FA, fractional anisotropy; MK, mean kurtosis; MMSE, mini-mental state examination; MoCA, Montreal cognitive assessment.

As showed in Figure 3, The FA and MK values of the left putamen (*r* = 0.490, *p* < 0.001; *r* = 0.498, *p* < 0.001), the MD value of the right middle temporal lobe (*r* = −0.622, *p* < 0.001), and the CBF value of the inferior temporal lobe (*r* = 0.574, *p* < 0.001) were strongly correlated with the MMSE scores. Moreover, the FA and MK values of the left putamen (*r* = 0.474, *p* < 0.001; *r* = 0.472, *p* < 0.001), the MD value of the right inferior temporal lobe (*r* = −0.609, *p* < 0.001), and the CBF value of the left inferior temporal lobe (*r* = 0.588, *p* < 0.001) were strongly correlated with the MoCA scores.

### Correlations between DKI parameters and CBF values

As illustrated in Table 3, a higher CBF was associated with a lower MD, higher FA, or higher MK. These associations were statistically significant in most ROIs, including the left frontal lobe [for CBF and FA: β(SE) = 0.9(0.3) × 10^–3^, *p* = 0.014], left parietal lobe [for CBF and MD: β(SE) = −3.3(2.0) × 10^–3^, *p* = 0.036; for CBF and FA: β(SE) = 0.6(0.2) × 10^–3^, *p* = 0.025], right parietal lobe [for CBF and MD: β(SE) = −3.2(0.9) × 10^–3^, *p* = 0.019; for CBF and MK: β(SE) = 1.3(0.6) × 10^–3^, *p* = 0.041], left temporal lobe [for CBF and MD: β(SE) = −5.5(2.0) × 10^–3^, *p* = 0.001], right temporal lobe [for CBF and MD: β(SE) = −5.1(0.7) × 10^–3^, *p* < 0.001; for CBF and FA: β(SE) = −0.7(0.2) × 10^–3^, *p* = 0.018], left occipital lobe [for CBF and MD: β(SE) = −4.9(0.9) × 10^–3^, *p* < 0.001], and right occipital lobe [for CBF and MD: β(SE) = −3.9(1.0) × 10^–3^, *p* = 0.004].

**TABLE 3:** Regression coefficient and significance in simple linear regression models between CBF and DKI metrics.

	CBF^a^		CBF^b^		CBF^c^	
**ROI**	**β (SE), ×10^–3^**	***p*-value**	**β (SE), ×10^–3^**	***p*-value**	**β (SE), ×10^–3^**	***p*-value**
**lh**						
FL	−1.8 (1.0)	0.165	0.9 (0.3)	**0.014**	1.9 (0.9)	0.061
PL	−3.3 (2.0)	**0.036**	0.6 (0.2)	**0.025**	1.4 (0.8)	0.088
TL	−5.5 (2.0)	**0.001**	0.09 (0.3)	0.748	0.4 (0.7)	0.56
OL	−4.9 (0.9)	**<0.001**	0.5 (0.3)	0.114	1.1 (0.7)	0.103
**rh**						
FL	−2.1 (1.0)	0.127	0.6 (0.3)	0.114	1.4 (1.0)	0.17
PL	−3.2 (0.9)	**0.019**	0.4 (0.2)	0.077	1.3 (0.6)	**0.041**
TL	−5.1 (0.7)	**<0.001**	0.7 (0.2)	**0.018**	0.9 (0.6)	0.12
OL	−3.9 (1.0)	**0.004**	0.3 (0.2)	0.284	0.05 (0.6)	0.939

Regression coefficient and significance adjusted for age and sex were determined after correction for a false discovery rate.

^a^The mean diffusivity is the dependent variable.

^b^The fractional anisotropy is the dependent variable.

^c^The mean kurtosis is the dependent variable. CBF, cerebral blood flow; FL, frontal lobes; OL, occipital lobes; PL, parietal lobes; TL, temporal lobes; ROI, region of interest; lh, left hemisphere; rh, right hemisphere.

The bold values mean *p* < 0.05 and significant correlation between CBF and DKI metrics.

### KNN analyses for diagnostic performance

**FIGURE 4:**
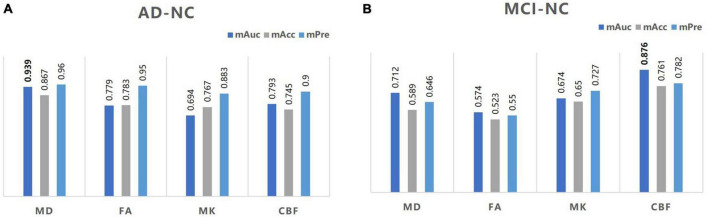
A bar graph of mAuc, mAcc, and mPre in the CBF and DKI metrics between the three groups. The mAuc, mAcc, and mPre data of the CBF and DKI metrics to distinguish the AD from the NC group **(A)** and the MCI from the NC group **(B)**. mAuc, mean area under the curve; mAcc, mean accuracy; mPre, mean precision.

An exploratory KNN analysis was undertaken using different MD, FA, MK, and CBF metrics to test the diagnostic performance of these diffusion and perfusion parameters. For distinguishing the AD group from the NC group, MD values performed best (mAuc = 0.939, mAcc = 0.867, mPre = 0.96), MK values performed worst (mAuc = 0.694, mAcc = 0.767, mPre = 0.883). For distinguishing the MCI group from the NC group, CBF values performed best (mAuc = 0.876, mAcc = 0.761, mPre = 0.782), FA values performed worst (mAuc = 0.574, mAcc = 0.523, mPre = 0.550) (Figure 4 and Supplementary Table 4).

## Discussion

This study assessed cortical microstructure and perfusion changes and their relationship in the AD continuum. In addition, the diagnostic efficacy of DKI and CBF parameters was explored. We found the following: (1) the MD values increased significantly, and the FA, MK, and CBF decreased significantly with AD progression, suggesting GM microstructural and perfusion abnormalities in both AD and MCI patients; (2) GM microstructural and perfusion changes are intimately related in AD, providing evidence of associations between regional changes in blood supply and cerebral tissue integrity; (3) the MD, FA, MK, and CBF values of many regions were significantly correlated with the MMSE and MoCA scores, demonstrating that cortical microstructural and blood flow abnormalities associate with cognitive decline; and (4) the CBF values had the best diagnostic power for distinguishing MCI from NC; MD values had the highest diagnostic ability to distinguish AD from NC.

Previous cross-sectional and longitudinal studies on CBF in AD demonstrated that reduced CBF served as an essential imaging biomarker of early disease detection and progression (Asllani et al., 2008; Weijs et al., 2022), and differences in CBF abnormalities were more sensitive than those noted with structural atrophy. In our study, the CBF in numerous regions decreased in AD and MCI patients compared to the NC group, as expected. Furthermore, our study confirmed that the CBF deficit was linked to cognitive impairment. These results were consistent with other AD perfusion reports (Binnewijzend et al., 2013; Ding et al., 2014), showing that CBF decreased gradually with the progression of AD. In addition, one study confirmed that Aβ constricted brain capillaries at pericyte locations, resulting in cerebral perfusion reduction (Nortley et al., 2019). Hence, decreased CBF is closely related to brain vasculature damage. CBF should be considered a potential target for prevention and treatment from a cardiovascular point of view in future research.

We found a significant elevation of MD in the cortical and deep GM. Consistent with previous studies (Rose et al., 2008; Gong et al., 2017), the changes in the cortical GM primarily occurred in the frontal and parietal cortices, including the superior frontal, caudal middle frontal, rostral middle frontal, medial orbital frontal, lateral orbital frontal, precentral gyrus, superior parietal, and inferior parietal lobule in the MCI group; also, the impairment regions were more extensive at the AD stage. MD can be used to examine differences in brain structural integrity. In the present study, increased MD in the cortex suggested early microstructure breakdown due to synaptic and dendritic loss and damage to cellular membranes that occur prior to the development of clinical dementia (Le Bihan, 2003).

As fewer barriers to diffusion appeared, molecules were able to diffuse more freely, and MD was generally expected to increase, implying a decline in FA that was positively associated with neuron density (Preziosa et al., 2019). Notably, we found MD was the most sensitive metric for capturing the loss of microstructural integrity in AD and MCI among the three DKI metrics, as well as serving as meaningful imaging biomarkers for assessing cognitive status. Rodriguez-Vieitez et al. (2021) suggested that higher cortical MD predicted faster hippocampal atrophy and clinical progression to MCI. Furthermore, the MK metric can complement conventional MD and FA for detecting microstructural changes and serving as diagnostic imaging biomarkers.

As with previous studies (Kantarci et al., 2005; Muller et al., 2005; Fellgiebel et al., 2006), the most significant microstructural abnormality reflected by MD in the deep GM were in the hippocampus, which was consistent with the neuropathological features of AD. Douaud et al. (2013) found that left hippocampal MD was the best predictor of future progression to AD. Interestingly, we found MK exhibited poor power at capturing microstructural differences in the deep GM, while previous studies have reported the advantages of diffusional kurtosis metrics in the deep GM (Pelizzari et al., 2019). However, some research has also shown that alterations in kurtosis parameters were limited in AD (Tu et al., 2021; Raj et al., 2022). Wang et al. (2018) found that alterations of MK in subcortical nuclei were weak in AD. We hypothesized that the microstructural changes were covered up by the volume loss. So, in order to comprehensively study the change pattern of microstructure in deep GM in cognitive impairments, future studies need to involve GM volume and microstructure simultaneously.

Robust correlations between DKI parameters, CBF, and cognitive scores were found in many regions, particularly in the MD value of the right inferior temporal lobe and the left hippocampus, verifying that cognition is related to changes in the GM microstructure. Evidence confirmed the association between the microstructural changes of hippocampus and cognitive scores (Shahid et al., 2022). A previous study showed inferior temporal tau burden were associated with activities of daily living impairment in AD (Halawa et al., 2019). Unlike our study, Gong et al. (2013) found an association between GM MD and the MMSE score in the frontal and parietal lobes. While our results were consistent with the sequence of pathology progression in AD (Berron et al., 2021).

We found that a lower CBF was associated with a higher MD, lower FA, and lower MK. These data supported an intimate relationship between tissue integrity and cerebral perfusion changes. In addition, this finding is consistent with the association between CBF and DTI metrics in a cross sectional study reported by Kiely et al. (2022). Meanwhile, a study also found that a positive association between hypoperfusion and white matter damage, and myelin integrity declines with CBF across cognitively normal subjects (Bouhrara et al., 2020). This pattern occurs whether regions are in GM or white matter, to some extent, it reveals that the blood supply offers some protection to cerebral structures. For the results of the article, it is possible that Aβ accumulation leads to vascular injuries, such as vascular wall destruction, pericyte contraction, or neurovascular unit uncoupling (Chakraborty et al., 2017; Sweeney et al., 2018; Apátiga-Pérez et al., 2022), and vascular damage leads to a further CBF reduction. Due to disruptive homeostasis between the blood supply and neuronal activity, damage to the synapses and neurons occurs. These results are biologically plausible, as described previously. In addition, we also found that CBF, MD, and FA changes coexisted in the parietal and temporal lobes. This progression pattern indicated that synergy effects between CBF and brain microstructure may occur early in AD. Therefore, it is essential to explore the extent of this potential association to understand the role of the blood supply and brain tissue integrity in AD.

By analyzing the diagnostic performance of DKI and CBF parameters using the KNN model, we found that the diagnostic value of MD was highest for AD and NC diagnoses, while the diagnostic value of CBF was best for MCI and NC diagnoses. A machine learning study (Collij et al., 2016) showed that CBF can be used to classify the diagnosis of AD disease stages with good accuracy. Furthermore, in the present study, we noted that GM microstructure had great diagnosis value, a finding yet to be extensively discussed in the literature. Some papers explored the predictive performance of hippocampal diffusivity. In a 3-year longitudinal study, Douaud et al. (2013) found that hippocampal diffusivity was more sensitive than white matter tracts and was also a better predictor than CSF Aβ and tau measurements. Similarly, a study suggested that hippocampal MD had high diagnostic accuracy in detecting dementia (Brueggen et al., 2018). The importance of cerebral microstructure should be considered in early disease detection.

This study had some limitations. A limited sample size and unbalanced groups resulted in some bias. Future research needs large-scale, multi-center studies to explore the underlying relationship between brain perfusion and diffusion. Then, we hope to conduct a longitudinal study to trace the trajectory of the cerebral microstructure and blood flow. Only with longitudinal comparisons can we confirm the dynamic performance of these changes with AD progression. Moreover, patients at the preclinical stage (i.e., subjective cognitive decline, SCD) should be enrolled in future studies. In this way, we would be able to confirm that these changes occur earlier and they could become a latent target for early diagnosis and treatment.

## Conclusion

In conclusion, this study showed that GM microstructure and CBF were closely related in AD. Increased MD, decreased FA, and MK were accompanied by decreased blood perfusion throughout the AD course. CBF and MD values help predict the AD and MCI diagnoses. These results should be considered in future clinical trials.

## Data availability statement

The original contributions presented in this study are included in the article/Supplementary material, further inquiries can be directed to the corresponding author.

## Ethics statement

The studies involving human participants were reviewed and approved by the Tianjin First Central Hospital Medical Ethics Committee. The patients/participants provided their written informed consent to participate in this study.

## Author contributions

XN and YG designed the study, collected the data, and wrote the manuscript. ZC contributed to the image processing. TL and YC contributed to the data analysis and interpretation. XZ contributed to the revision of the manuscript. HN participated in the design of the experiment and provided the main idea for the manuscript. All authors contributed to the article and approved the submitted version.

## Footnotes

1
http://www.cereflow.cn

2
http://fsl.fmrib.ox.ac.uk/fsl/fslwiki/

3
https://www.nitrc.org/projects/dke

4
http://surfer.nmr.mgh.harvard.edu/
