# Supplementary material for: The correlation between changes in gray matter microstructure and cerebral blood flow in Alzheimer’s disease
Source: Front Aging Neurosci. 2023 Jun 2;15:1205838. doi: 10.3389/fnagi.2023.1205838 (PMC10272452; doi:10.3389/fnagi.2023.1205838)
Supplement: Supplementary file 1 [file Data_Sheet_1.docx]

Supplementary Material

**The correlation between** **changes in gray matter microstructure and cerebral blood flow in Alzheimer’s disease**

**Xiaoxi Niu^1†^, Ying Guo^1†^, Zhongyu Chang^2^, Tongtong Li^3^, Yuanyuan Chen^4^, Xianchang Zhang^5^, Hongyan Ni^6*^**

^1^Department of Radiology, Tianjin First Central Hospital, Tianjin Medical University, Tianjin, China

^2^Department of Radiology and Tianjin Key Laboratory of Functional Imaging, Tianjin Medical University General Hospital, Tianjin, China

^3^Department of Radiology, The Affiliated Hospital of Hebei University, Baoding, China

^4^Tianjin International Joint Research Center for Neural Engineering, Academy of Medical Engineering and Translational Medicine, Tianjin University, Tianjin, China

^5^MR Collaboration, Siemens Healthcare Ltd., Beijing, China

^6^Department of Radiology, Tianjin First Central Hospital, Tianjin, China

*** Correspondence:**

Hongyan Ni

[hongyni@163.com](mailto:hongyni@163.com)

**Table 1. A comparison of CBF and DKI-derived parameters in the cortical gray matter of the MCI patients vs. the NC subjects**

|  | Region | Side | Size(mm^2^) | x | y | z | Clusterwise p-value |
| --- | --- | --- | --- | --- | --- | --- | --- |
| CBF | inferior temporal | LH | 4363.44 | -56.9 | -48.5 | -17.1 | 0.00200 |
|  | isthmus cingulate | LH | 833.11 | -5.5 | -43.0 | 20.5 | 0.00200 |
|  | lateral occipital | LH | 739.41 | -18.7 | -93.4 | -9.8 | 0.00200 |
|  | superior parietal | LH | 484.40 | -26.0 | -63.0 | 30.2 | 0.00599 |
|  | caudal middle frontal | LH | 438.22 | -37.9 | 11.1 | 36.0 | 0.00599 |
|  | inferior temporal | RH | 5053.50 | 44.5 | -51.8 | -11.7 | 0.00200 |
|  | isthmus cingulate | RH | 1191.45 | 6.2 | -48.7 | 29.1 | 0.00200 |
|  | inferior parietal | RH | 309.18 | 33.8 | -68.9 | 37.8 | 0.01594 |
|  | caudal middle frontal | RH | 177.78 | 36.8 | 18.1 | 29.1 | 0.03568 |
| MD | precuneus | LH | 5175.51 | -4.6 | -63.3 | 25.5 | 0.00200 |
|  | superior parietal | LH | 1517.26 | -20.0 | -64.2 | 36.9 | 0.00200 |
|  | pars opercularis | LH | 1263.90 | -37.3 | 15.8 | 10.1 | 0.00200 |
|  | entorhinal | LH | 1250.14 | -24.8 | -9.9 | -33.7 | 0.00200 |
|  | caudal middle frontal | LH | 258.03 | -28.8 | 5.2 | 52.8 | 0.00599 |
|  | isthmus cingulate | LH | 203.31 | -14.3 | -42.2 | -0.5 | 0.01594 |
|  | middle temporal | RH | 7941.94 | 59.6 | -51.6 | 4.7 | 0.00200 |
|  | rostral middle frontal | RH | 2079.52 | 33.6 | 27.4 | 44.3 | 0.00200 |
|  | superior frontal | RH | 1255.79 | 21.2 | -4.4 | 56.2 | 0.00200 |
|  | fusiform | RH | 866.48 | 35.2 | -14.0 | -33.0 | 0.00200 |
|  | pars opercularis | RH | 832.20 | 44.4 | 8.2 | 7.2 | 0.00200 |
|  | postcentral | RH | 776.50 | 24.8 | -36.6 | 55.1 | 0.00200 |
|  | superior parietal | RH | 414.84 | 24.9 | -79.5 | 19.8 | 0.00200 |
|  | precuneus | RH | 405.62 | 14.8 | -44.9 | 35.2 | 0.00200 |
|  | precentral | RH | 315.74 | 8.2 | -24.0 | 73.5 | 0.00798 |
|  | medial orbitofrontal | RH | 287.08 | 5.5 | 22.4 | -21.0 | 0.00798 |
|  | inferior parietal | RH | 199.53 | 41.5 | -74.8 | 13.0 | 0.03568 |
|  | lateral orbitofrontal | RH | 191.13 | 15.1 | 22.1 | -14.7 | 0.03568 |
| FA | lateral orbitofrontal | LH | 712.47 | -42.3 | 26.8 | -13.9 | 0.00200 |
|  | precentral | RH | 978.30 | 56.5 | 0.3 | 36.1 | 0.00200 |
|  | superior temporal | RH | 235.15 | 65.1 | -18.7 | 1.3 | 0.03174 |
| MK | precuneus | RH | 453.65 | 9.1 | -56.5 | 14.8 | 0.01395 |
|  | fusiform | RH | 257.42 | 34.7 | -17.3 | -29.5 | 0.04156 |

CBF, cerebral blood flow; MD, mean diffusivity; FA, fractional anisotropy; MK, mean kurtosis; LH, left hemisphere; RH, right hemisphere; MCI, mild cognitive impairment; NC, normal control. Cluster-wise p-value was generated after cluster correction (p < 0.05) in FreeSurfer software.

**Table 2. A comparison of CBF and DKI-derived parameters in the cortical gray matter of the AD patients vs. the NC subjects**

|  | Region | Side | Size(mm^2^) | x | y | z | Clusterwise p-value |
| --- | --- | --- | --- | --- | --- | --- | --- |
| CBF | inferior temporal | LH | 15952.19 | -42.7 | -48.7 | -12.6 | 0.00200 |
|  | caudal middle frontal | LH | 4219.06 | -39.7 | 9.0 | 46.0 | 0.00200 |
|  | lateral orbitofrontal | LH | 2300.38 | -22.2 | 32.9 | -10.4 | 0.00200 |
|  | isthmus cingulate | LH | 1971.92 | -10.0 | -39.3 | 33.1 | 0.00200 |
|  | medial orbitofrontal | LH | 396.67 | -7.7 | 34.8 | -16.9 | 0.00200 |
|  | precentral | LH | 335.97 | -33.1 | -16.3 | 44.3 | 0.00599 |
|  | parahippocampal | LH | 268.43 | -24.5 | -23.6 | -26.6 | 0.00997 |
|  | caudal anterior cingulate | LH | 246.58 | -8.6 | 23.3 | 26.7 | 0.01395 |
|  | entorhinal | LH | 179.41 | -28.5 | -12.6 | -32.0 | 0.02188 |
|  | superior temporal | LH | 163.38 | -59.9 | -48.6 | 14.2 | 0.02780 |
|  | lateral occipital | LH | 134.29 | -11.4 | -98.7 | 2.0 | 0.03371 |
|  | precuneus | RH | 3069.03 | 4.8 | -58.6 | 24.6 | 0.00200 |
|  | medial orbitofrontal | RH | 2753.18 | 15.2 | 42.0 | 1.6 | 0.00200 |
|  | rostral middle frontal | RH | 881.27 | 28.1 | 37.2 | 20.8 | 0.00200 |
|  | inferior parietal | RH | 799.00 | 32.7 | -66.7 | 29.7 | 0.00200 |
|  | rostral middle frontal | RH | 708.50 | 42.2 | 20.1 | 31.4 | 0.00200 |
|  | temporal pole | RH | 663.32 | 32.0 | 13.5 | -36.8 | 0.00200 |
|  | inferior temporal | RH | 660.82 | 53.7 | -30.6 | -17.6 | 0.00200 |
|  | pericalcarine | RH | 301.57 | 13.1 | -95.0 | 7.0 | 0.01990 |
|  | lateral orbitofrontal | RH | 285.40 | 32.6 | 41.2 | -9.4 | 0.02188 |
|  | parahippocampal | RH | 280.78 | 22.8 | -25.0 | -20.6 | 0.02386 |
| MD | precuneus | LH | 19507.87 | -18.3 | -58.6 | 18.2 | 0.00200 |
|  | caudal middle frontal | LH | 2482.38 | -26.8 | -1.1 | 46.2 | 0.00200 |
|  | inferior parietal | LH | 2190.57 | -39.7 | -54.4 | 20.9 | 0.00200 |
|  | rostral middle frontal | LH | 1702.28 | -34.3 | 34.1 | 12.4 | 0.00200 |
|  | insula | LH | 1698.32 | -28.0 | 23.4 | 5.4 | 0.00200 |
|  | superior parietal | LH | 1568.30 | -20.2 | -64.3 | 36.0 | 0.00200 |
|  | precentral | LH | 681.04 | -50.4 | -5.1 | 27.8 | 0.00200 |
|  | middle temporal | LH | 346.28 | -57.7 | -18.8 | -24.0 | 0.01990 |
|  | supramarginal | LH | 306.32 | -43.0 | -52.6 | 37.7 | 0.02583 |
|  | inferior temporal | RH | 39797.78 | 52.8 | -31.0 | -18.4 | 0.00200 |
|  | superior parietal | RH | 2904.58 | 26.4 | -76.6 | 18.2 | 0.00200 |
|  | medial orbitofrontal | RH | 1322.93 | 5.6 | 21.9 | -20.1 | 0.00200 |
|  | lateral occipital | RH | 467.35 | 34.1 | -78.2 | 8.6 | 0.00200 |
| FA | entorhinal | LH | 229.46 | -25.3 | -6.7 | -31.7 | 0.00200 |
|  | posterior cingulate | LH | 185.80 | -4.7 | -20.9 | 27.9 | 0.00200 |
|  | superior parietal | LH | 142.02 | -21.7 | -62.6 | 33.7 | 0.00200 |
|  | inferior parietal | LH | 127.40 | -37.0 | -52.0 | 33.3 | 0.00200 |
|  | precuneus | LH | 114.59 | -18.3 | -41.2 | 45.5 | 0.00200 |
|  | precentral | RH | 1236.05 | 55.9 | 1.6 | 34.3 | 0.00200 |
|  | pars triangularis | RH | 555.83 | 48.9 | 34.7 | 0.8 | 0.00200 |
|  | rostral middle frontal | RH | 401.61 | 25.8 | 32.3 | 33.6 | 0.00200 |
|  | superior frontal | RH | 234.56 | 21.2 | -6.3 | 54.7 | 0.00200 |
|  | middle temporal | RH | 64.11 | 58.0 | -29.3 | -16.5 | 0.02977 |
| MK | superior temporal | LH | 851.26 | -51.1 | -36.6 | 9.6 | 0.00200 |
|  | caudal anterior cingulate | LH | 730.65 | -10.3 | 19.8 | 29.5 | 0.00200 |
|  | temporal pole | LH | 577.49 | -31.5 | 13.0 | -35.6 | 0.00200 |
|  | pars opercularis | LH | 322.40 | -41.9 | 23.1 | 19.3 | 0.02386 |
|  | precuneus | RH | 1019.57 | 18.2 | -55.2 | 18.8 | 0.00200 |
|  | lateral occipital | RH | 716.00 | 29.4 | -83.1 | 3.3 | 0.00200 |
|  | caudal middle frontal | RH | 677.73 | 34.1 | 15.8 | 29.3 | 0.00200 |
|  | insula | RH | 641.85 | 36.9 | -9.2 | -9.7 | 0.00200 |
|  | precentral | RH | 589.02 | 43.9 | -11.8 | 28.9 | 0.00400 |
|  | inferior parietal | RH | 467.65 | 42.9 | -59.0 | 20.0 | 0.00599 |
|  | superior temporal | RH | 428.20 | 46.7 | -18.5 | -12.6 | 0.00997 |
|  | rostral middle frontal | RH | 387.42 | 37.2 | 39.9 | 13.3 | 0.01196 |
|  | pericalcarine | RH | 294.26 | 13.1 | -95.0 | 7.0 | 0.04352 |

CBF, cerebral blood flow; MD, mean diffusivity; FA, fractional anisotropy; MK, mean kurtosis; LH, left hemisphere; RH, right hemisphere; NC, normal control; AD, Alzheimer’s disease. Cluster-wise p-value was generated after cluster correction (p < 0.05) in FreeSurfer software.

**Table 3. A comparison of CBF and DKI-derived parameters in the cortical gray matter of the MCI patients vs. the AD subjects**

|  | Region | Side | Size(mm^2^) | x | y | z | Clusterwise p-value |
| --- | --- | --- | --- | --- | --- | --- | --- |
| CBF | lateral occipital | LH | 2075.31 | -24.1 | -96.8 | 0.6 | 0.00200 |
|  | superior temporal | LH | 514.10 | -46.0 | -41.0 | 16.3 | 0.00400 |
|  | inferior temporal | LH | 436.99 | -57.7 | -46.7 | -18.3 | 0.00599 |
|  | lingual | LH | 381.96 | -11.1 | -67.9 | 2.5 | 0.00599 |
|  | supramarginal | LH | 311.20 | -40.9 | -43.4 | 38.4 | 0.00798 |
|  | pars opercularis | LH | 197.32 | -45.7 | 15.7 | 8.6 | 0.01594 |
|  | inferior parietal | LH | 173.38 | -34.1 | -59.0 | 37.4 | 0.02188 |
|  | precentral | LH | 172.50 | -32.9 | -11.0 | 52.4 | 0.02188 |
|  | caudal middle frontal | LH | 109.62 | -34.6 | 2.7 | 31.5 | 0.04742 |
|  | fusiform | RH | 5877.80 | 40.6 | -54.3 | -13.0 | 0.00200 |
|  | middle temporal | RH | 755.52 | 53.6 | -2.4 | -30.7 | 0.00200 |
|  | lingual | RH | 264.10 | 14.2 | -62.0 | 2.4 | 0.00798 |
| MD | fusiform | LH | 10323.78 | -31.7 | -35.4 | -23.9 | 0.00200 |
|  | posterior cingulate | LH | 2137.59 | -3.8 | -19.3 | 34.6 | 0.00200 |
|  | lingual | LH | 927.19 | -21.0 | -65.8 | 0.5 | 0.00200 |
|  | rostral middle frontal | LH | 674.84 | -40.9 | 24.2 | 19.1 | 0.00200 |
|  | superior frontal | LH | 590.57 | -19.9 | 26.9 | 37.3 | 0.00200 |
|  | inferior temporal | RH | 11267.25 | 52.6 | -31.9 | -18.5 | 0.00200 |
|  | insula | RH | 3261.83 | 35.5 | -6.8 | -6.2 | 0.00200 |
|  | rostral middle frontal | RH | 2937.72 | 36.4 | 38.9 | 12.9 | 0.00200 |
|  | precuneus | RH | 1707.32 | 12.0 | -56.9 | 20.6 | 0.00200 |
|  | caudal middle frontal | RH | 634.85 | 24.9 | -0.8 | 45.8 | 0.00200 |
|  | inferior parietal | RH | 545.47 | 33.1 | -68.5 | 27.5 | 0.00200 |
|  | fusiform | RH | 330.17 | 38.4 | -19.8 | -27.1 | 0.01196 |
| FA | rostral middle frontal | LH | 615.88 | -40.1 | 23.6 | 19.9 | 0.00200 |
|  | temporal pole | LH | 505.66 | -31.0 | 13.4 | -37.6 | 0.00200 |
|  | supramarginal | LH | 274.01 | -44.2 | -54.1 | 38.8 | 0.02583 |
|  | superior parietal | LH | 249.41 | -24.7 | -65.2 | 26.9 | 0.03371 |
|  | lateral occipital | RH | 1133.45 | 38.9 | -82.8 | -13.1 | 0.00200 |
|  | rostral middle frontal | RH | 441.59 | 36.7 | 39.6 | 11.9 | 0.00798 |
|  | postcentral | RH | 415.92 | 48.3 | -12.6 | 28.6 | 0.01196 |
|  | caudal middle frontal | RH | 404.50 | 32.5 | 12.1 | 29.3 | 0.01196 |
|  | precuneus | RH | 244.60 | 17.9 | -55.0 | 17.6 | 0.04352 |
| MK | posterior cingulate | LH | 407.21 | -4.1 | -24.2 | 27.0 | 0.01990 |

CBF, cerebral blood flow; MD, mean diffusivity; FA, fractional anisotropy; MK, mean kurtosis; LH, left hemisphere; RH, right hemisphere; MCI, mild cognitive impairment; AD, Alzheimer’s disease. Clusterwise p-value was generated after cluster correction (p < 0.05) in FreeSurfer software.

**Table 4. Performance of all parameters with KNN model.**

| group | parameters | mAuc | mAcc | mPre |
| --- | --- | --- | --- | --- |
| AD/NC | CBF | 0.793 | 0.745 | 0.9 |
|  | MD | 0.939 | 0.867 | 0.96 |
|  | FA | 0.779 | 0.783 | 0.95 |
|  | MK | 0.694 | 0.767 | 0.883 |
| MCI/NC | CBF | 0.876 | 0.761 | 0.782 |
|  | MD | 0.712 | 0.598 | 0.646 |
|  | FA | 0.574 | 0.523 | 0.550 |
|  | MK | 0.674 | 0.65 | 0.727 |

NC, normal control; AD, Alzheimer’s disease; MCI, mild cognitive impairment; KNN, K-Nearest Neighbor; CBF, cerebral blood flow; MD, mean diffusivity; FA, fractional anisotropy; MK, mean kurtosis; mAuc, mean the area under the receiver operating characteristic curve; mAcc, mean accuracy; mPre, mean precision.
